# Effect of tamoxifen and radiotherapy in women with locally excised ductal carcinoma in situ: long-term results from the UK/ANZ DCIS trial

**DOI:** 10.1016/S1470-2045(10)70266-7

**Published:** 2010-12-08

**Authors:** Jack Cuzick, Ivana Sestak, Sarah E Pinder, Ian O Ellis, Sharon Forsyth, Nigel J Bundred, John F Forbes, Hugh Bishop, Ian S Fentiman, William D George

**Affiliations:** a Cancer Research UK, Centre for Epidemiology, Mathematics, and Statistics, Wolfson Institute of Preventive Medicine, Queen Mary School of Medicine and Dentistry, University of London, London, UK; b Division of Cancer Studies, Research Oncology, King's College London, Guy's Hospital, London, UK; c Academic Oncology, Guy's Hospital, London, UK; d City Hospital, Department of Histopathology, Nottingham, UK; e Cancer Research UK and UCL Clinical Trials Centre, Department of Oncology, London, UK; f South Manchester University Hospital, Department of Surgery, Manchester, UK; g School of Medical Practice and Population Health, University of Newcastle, Newcastle, NSW, Australia; h Beaumont Hospital, Bolton, UK; i Western Infirmary Glasgow, Glasgow, UK

## Abstract

**Background:**

Initial results of the UK/ANZ DCIS (UK, Australia, and New Zealand ductal carcinoma in situ) trial suggested that radiotherapy reduced new breast events of ipsilateral invasive and ductal carcinoma in situ (DCIS) compared with no radiotherapy, but no significant effects were noted with tamoxifen. Here, we report long-term results of this trial.

**Methods:**

Women with completely locally excised DCIS were recruited into a randomised 2×2 factorial trial of radiotherapy, tamoxifen, or both. Randomisation was independently done for each of the two treatments (radiotherapy and tamoxifen), stratified by screening assessment centre, and blocked in groups of four. The recommended dose for radiation was 50 Gy in 25 fractions over 5 weeks (2 Gy per day on weekdays), and tamoxifen was prescribed at a dose of 20 mg daily for 5 years. Elective decision to withhold or provide one of the treatments was permitted. The endpoints of primary interest were invasive ipsilateral new breast events for the radiotherapy comparison and any new breast event, including contralateral disease and DCIS, for tamoxifen. Analysis of each of the two treatment comparisons was restricted to patients who were randomly assigned to that treatment. Analyses were by intention to treat. All trial drugs have been completed and this study is in long-term follow-up. This study is registered, number ISRCTN99513870.

**Findings:**

Between May, 1990, and August, 1998, 1701 women were randomly assigned to radiotherapy and tamoxifen, radiotherapy alone, tamoxifen alone, or to no adjuvant treatment. Seven patients had protocol violations and thus 1694 patients were available for analysis. After a median follow-up of 12·7 years (IQR 10·9–14·7), 376 (163 invasive [122 ipsilateral *vs* 39 contralateral], 197 DCIS [174 ipsilateral *vs* 17 contralateral], and 16 of unknown invasiveness or laterality) breast cancers were diagnosed. Radiotherapy reduced the incidence of all new breast events (hazard ratio [HR] 0·41, 95% CI 0·30–0·56; p<0·0001), reducing the incidence of ipsilateral invasive disease (0·32, 0·19–0·56; p<0·0001) as well as ipsilateral DCIS (0·38, 0·22–0·63; p<0·0001), but having no effect on contralateral breast cancer (0·84, 0·45–1·58; p=0·6). Tamoxifen reduced the incidence of all new breast events (HR 0·71, 95% CI 0·58–0·88; p=0·002), reducing recurrent ipsilateral DCIS (0·70, 0·51–0·86; p=0·03) and contralateral tumours (0·44, 0·25–0·77; p=0·005), but having no effect on ipsilateral invasive disease (0·95, 0·66–1·38; p=0·8). No data on adverse events except cause of death were collected for this trial.

**Interpretation:**

This updated analysis confirms the long-term beneficial effect of radiotherapy and reports a benefit for tamoxifen in reducing local and contralateral new breast events for women with DCIS treated by complete local excision.

**Funding:**

Cancer Research UK and the Australian National Health and Medical Research Council.

## Introduction

Ductal carcinoma in situ (DCIS) is usually an asymptomatic disorder that is characterised by a clonal proliferation of epithelial cells confined within the lumen of mammary ducts.^1^, ^2^ Screening has led to a substantial increase in the incidence of DCIS over the past two decades; the disorder represents 10% of all breast carcinomas and around 20% of screen-detected cancers.^3^, ^4^, ^5^

Management options for DCIS include surgery, radiotherapy, and hormonal therapy.^6^ The effectiveness of radiotherapy in reducing recurrences has been examined in four clinical trials.^7^, ^8^, ^9^, ^10^, ^11^ In all these studies, radiotherapy reduced in-situ or invasive recurrences by about 50%. Although radiotherapy is associated with substantial reductions in local recurrence, no differences have been reported in metastatic disease or overall survival.^12^, ^13^

The role of tamoxifen in the management of DCIS has been investigated in the UK, Australia, and New Zealand (UK/ANZ) DCIS trial^10^ and also in the National Surgical Adjuvant Breast and Bowel Project B-24 (NSABP B-24) trial.^14^ In the NSABP trial, patients with DCIS received radiotherapy and were then randomised to tamoxifen (20 mg/day) or placebo. After just over 6 years of follow-up, a significant reduction in all new breast events was reported in the tamoxifen group compared with the placebo group (rate ratio 0·63, 95% CI 0·47–0·83; p=0·0009). The use of other endocrine treatments for DCIS is under investigation in the International Breast Cancer Intervention Study II (IBIS-II) and the NSABP B-35 trial.^15^

The UK/ANZ DCIS trial was a 2×2 factorial randomised trial that assessed radiotherapy, tamoxifen, or both in patients with completely excised DCIS.^10^ After a median follow-up of 4·4 years (range 0·2–9·9), patients who had radiotherapy had a lower incidence of ipsilateral invasive disease (hazard ratio [HR] 0·45, 95% CI 0·24–0·85) and ipsilateral DCIS (0·36, 0·19–0·66) than those who did not have radiotherapy, but there was no difference in contralateral disease between groups. Tamoxifen was weakly associated with a reduction in all new breast events compared with no tamoxifen (HR 0·83, 95% CI 0·64–1·06), but this was because of a reduction in all DCIS (0·68, 0·49–0·96); no reduction in invasive cancer was reported (1·11, 0·76–1·63). Here, we report updated results of the UK/ANZ DCIS trial with a median follow-up of 12·7 years.

## Methods

### Patients

Patients with unilateral or bilateral DCIS (>90% detected in the national breast screening programme) who were deemed suitable for breast conservation were entered into the trial. Patients with lobular carcinoma in situ, atypical ductal hyperplasia in the absence of DCIS, Paget's disease of the nipple, and those in whom pathological margins of the disease were uncertain were excluded, as were patients with a reduced life expectancy.

Patients were included if their lesion or lesions could be completely excised, which was confirmed by radiology of the surgical specimen and free margins on histological examination. If DCIS extended to the margin of the specimen, re-excision was done to establish clear margins. The size of lesion, pathological type of disease, and if re-excision was done was recorded, and an estimate of the maximum diameter of the total lesion was made. On an individual basis, surgeons in discussion with each patient decided whether to enter the patient into the four-way 2×2 randomisation, or one of two separate two-way randomisations with elective choice for the other treatment modality.

Patients were given information that described the disease, treatment options, and trial design. Patients provided written or verbal consent witnessed by a third party before entry into the trial. Ethics approval was obtained from local ethics committees at all participating hospitals.

### Randomisation and masking

Randomisation was done in one of three central trials offices by fax or telephone contact. Randomisation was independently done for each of the two treatments (radiotherapy and tamoxifen), stratified by screening assessment centre, and blocked in groups of four. The central trial centres each prepared their own randomisation lists using a common algorithm, and these lists were available only to trial staff who were trained in the randomisation procedure. Tamoxifen and radiotherapy were both given open label.

### Procedures

The recommended dose for radiotherapy was 50 Gy in 25 fractions over 5 weeks (2 Gy per day on weekdays; tumour dose fractionation 82) at the isocentre or at the point of intersection of the two tangential fields. We did not recommend boost treatment at the excision site. Tamoxifen was prescribed at a dose of 20 mg daily for 5 years.

Yearly bilateral mammography was recommended for the first 7 years after treatment and every 2 years thereafter. Dates and sites of histologically confirmed local new breast events (DCIS or invasive cancer), diagnosis of any new non-breast malignant disease, and causes of death were recorded. Patients in the UK who were lost to follow-up were registered with the Office for National Statistics (now the NHS Information Centre) to find out details of death (date and cause) and cancer registrations (date of registration).

The endpoints of primary interest were invasive ipsilateral new breast events for radiotherapy and any new breast event, including contralateral disease and DCIS for tamoxifen. We also individually assessed the effects of tamoxifen or radiotherapy on both recurrent DCIS or invasive ipsilateral breast cancer and contralateral DCIS or invasive cancer. We also measured distant new breast events as a first event, death after new breast events (breast cancer death), and cause-specific mortality. No data on adverse events were routinely collected, except for cause of death.

### Statistical analysis

To assess the effects of the two main treatment comparisons, analysis was restricted to patients who were randomly assigned to each main treatment comparison. Hence, for the tamoxifen comparison only patients who were randomly assigned to receive tamoxifen or not were included in the analysis. In the same way, patients who chose to receive radiotherapy or not were excluded from the main radiotherapy comparison. Thus, treatment comparisons were not confounded by the alternate treatment. All analyses were stratified according to whether or not patients received the alternate treatment, and whether this was by choice or as a result of random treatment allocation. Only the first new breast event was recorded; thus, for example, a distant new breast event that occurred after a local new breast event was not available for analysis.

Analyses were by intention to treat. We compared groups by the Cox proportional hazard model to estimate HRs, 95% CIs and two-sided p values. 10-year estimates and time-to-recurrence curves were produced with the Kaplan-Meier method. A p value of less than or equal to 0·05 was deemed significant. All calculations were done using Stata software (version 10.1).

This study is registered as an International Standard Randomised Controlled Trial, number ISRCTN99513870.

### Role of the funding source

The trial was developed by the breast cancer subcommittee of the UKCCCR (United Kingdom Coordinating Committee on Cancer Research; now the National Cancer Research Institute [NCRI]) and done through three trial offices (Cancer Research UK and University College London Cancer Trials Centre, London, UK; the Scottish Cancer Therapy Network, Edinburgh, UK; and the Australia and New Zealand Cancer Trials Office, Newcastle, NSW, Australia). The independent statistician (JC) had full access to all the data in the study and was responsible for providing regular information to the independent data monitoring committee. All authors were responsible for data interpretation, writing of the report, and final approval of the manuscript for submission. The corresponding author had final responsibility for the decision to submit for publication. The funding source had no role in the decision to publish this report.

## Results

**Figure 1:**
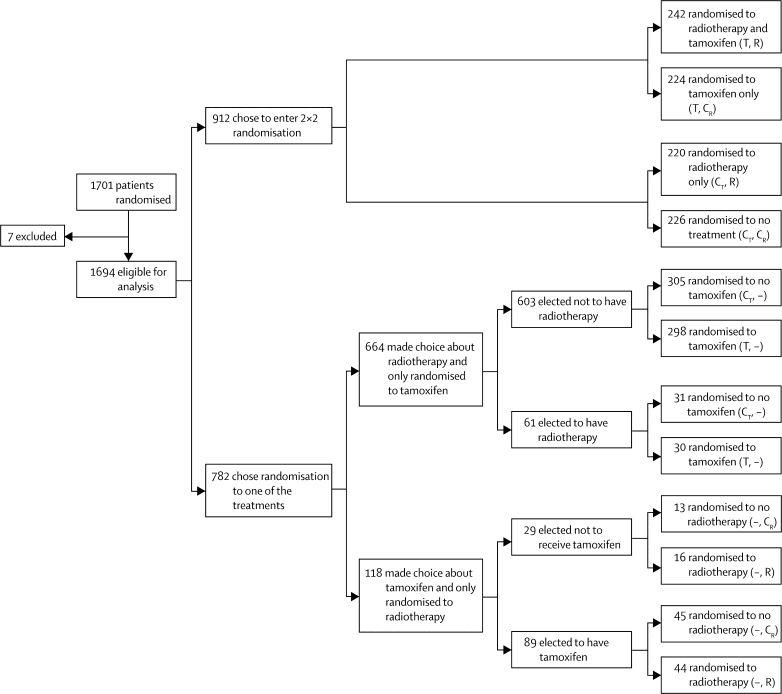
Trial profile T=tamoxifen. R=radiotherapy. C_T_=control group for tamoxifen. C_R_=control group for radiotherapy.

Between May, 1990, and August, 1998, 1701 patients were randomised in the UK DCIS trial (879 from the UK Trials Centre, 635 from the Scottish Trials Centre, and 187 from the Australia and New Zealand Trials Centre). After randomisation, seven patients were excluded from the analysis because of protocol violations; two patients had previous malignant disease, four patients underwent mastectomy before randomisation, and one patient had invasive cancer rather than DCIS. 59 patients had microinvasive disease and 130 patients were on hormone replacement therapy at time of randomisation. Because neither factor was confounding, these patients were included in the analysis. Thus, 1694 patients were eligible for analysis (figure 1).

Follow-up was complete up to Oct 1, 2008, and events after that time are not included in this analysis. Median follow-up was 12·7 years (IQR 10·9–14·7). 1363 (80%) of 1694 patients were 50–64 years old and 160 (9%) were under 50 years old at randomisation; these patients had either symptomatic or mammographically detected DCIS.

912 patients (54%) chose to enter the 2×2 randomisation (figure 1). 782 patients chose the two-way randomisation: 664 patients (39%) were randomly assigned to receive tamoxifen or not, of whom 603 elected not to receive radiotherapy and 61 received elective radiotherapy; 118 patients (7%) were randomly assigned to receive radiotherapy or not, of whom 29 elected not to receive tamoxifen and 89 received elective tamoxifen.

376 (22·2%) women had new breast events during the follow-up period (table 1): 197 (12%) patients had ductal carcinoma in situ (DCIS), 163 (10%) had invasive cancers, and the type of new breast events was unknown in 16 (1%).

**Table 1:** New breast events

			**No adjuvant treatment (n=544)**	**Tamoxifen alone (n=567)**	**Radiotherapy alone (n=267)**	**Radiotherapy and tamoxifen (n=316)**	**Total (n=1694)**
Follow-up (woman-years)			5428	6017	3023	3545	18 013
Breast events			174 (32%)	135 (24%)	35 (13%)	32 (10%)	376 (22%)
	DCIS		96 (18%)	72 (13%)	16 (6%)	13 (4%)	197 (12%)
		Ipsilateral	86 (16%)	63 (11%)	14 (5%)	11 (3%)	174 (10%)
		Contralateral	9 (2%)	4 (1%)	2 (1%)	2 (1%)	17 (1%)
		Unknown	1	5	0	0	6
	Invasive		72 (13%)	57 (10%)	16 (6%)	18 (6%)	163 (10%)
		Ipsilateral	52 (10%)	49 (9%)	10 (4%)	11 (3%)	122 (7%)
		Contralateral	20 (4%)	7 (1%)	5 (2%)	7 (2%)	39 (2%)
		Unknown	0	1	1	0	2
	Unknown		6 (1%)	6 (1%)	3 (1%)	1 (0%)	16 (1%)
Annual rate of breast events (%)			3·2%	2·2%	1·2%	0·9%	2·1%

Data are number (%). DCIS=ductal carcinoma in situ.

**Figure 2:**
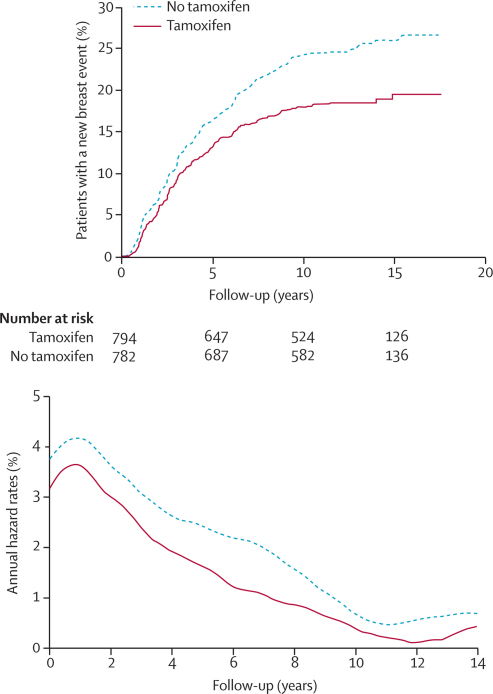
Kaplan-Meier curve for cumulative incidence and annual hazard plot of all breast events in the tamoxifen comparison

In total, 1576 patients were randomly assigned to receive tamoxifen or not (figure 1). 342 of these women developed a new breast event: 188 DCIS and 154 an invasive carcinoma (table 2). Fewer new breast events occurred in the patients randomly assigned to receive tamoxifen than in those who did not receive the drug (p=0·002; table 2; figure 2). Tamoxifen significantly reduced the rate of recurrent ipsilateral DCIS but not ipsilateral invasive disease (table 2). An absolute 10-year reduction of 3·9% was reported for all ipsilateral events. There was a significant reduction in all contralateral events in those randomly assigned to tamoxifen compared with those assigned to no tamoxifen (p=0·005; table 2), with an absolute 10-year reduction of 2·3%. Tamoxifen was associated with a reduction in the incidence of contralateral invasive events and there was weak evidence of a reduction in incidence of DCIS (table 2). Overall, an absolute 10-year reduction of 6·5% for all new breast events was achieved with the use of tamoxifen.

**Table 2:** New breast events and 10-year estimates of percentages with an event in patients randomised to tamoxifen or not

		**Randomised to tamoxifen (n=794)**	**Randomised to no tamoxifen (n=782)**	**Hazard ratio (95% CI)**	**p value**
**All patients**					
Ipsilateral		129 (15·7%)	162 (19·6%)	0·78 (0·62–0·99)	0·04
	Invasive	56 (6·8%)	60 (6·9%)	0·95 (0·66–1·38)	0·79
	DCIS	70 (8·6%)	97 (12·1%)	0·70 (0·51–0·86)	0·03
	Unknown	3	5	..	..
Contralateral		17 (1·9%)	38 (4·2%)	0·44 (0·25–0·77)	0·005
	Invasive	12 (1·5%)	25 (2·7%)	0·47 (0·24–0·94)	0·03
	DCIS	4 (0·3%)	11 (1·3%)	0·36 (0·11–1·12)	0·08
	Unknown	1	2	..	..
All invasive		69 (8·5%)	85 (9·1%)	0·81 (0·59–1·12)	0·2
All DCIS		77 (9·2%)	111 (13·6%)	0·67 (0·50–0·90)	0·008
All unknown*		5	8	..	..
All new breast events		151 (18·1%)	204 (24·6%)	0·71 (0·58–0·88)	0·002
**Patients not receiving radiotherapy (n=1053)**					
Ipsilateral		109 (13·2%)	140 (17·0%)	0·77 (0·59–0·98)	0·04
	Invasive	46 (5·5%)	51 (6·0%)	0·89 (0·59–1·33)	0·6
	DCIS	60 (7·4%)	84 (10·4%)	0·71 (0·51–0·99)	0·04
	Unknown	3	5	..	..
Contralateral		8 (0·9%)	29 (3·1%)	0·27 (0·12–0·59)	0·001
	Invasive	6 (0·8%)	20 (2·0%)	0·29 (0·12–0·73)	0·009
	DCIS	2 (0·0%)	9 (1·0%)	0·22 (0·05–1·01)	0·05
	Unknown	0	0	..	..
All unknown†		5	2	..	..
All new breast events		122 (14·6%)	171 (20·7%)	0·71 (0·57–0·87)	0·001
**Patients receiving radiotherapy (n=523)**					
Ipsilateral		20 (2·4%)	22 (2·6%)	0·93 (0·50–1·75)	0·8
	Invasive	10 (1·3%)	9 (0·9%)	1·41 (0·54–3·70)	0·5
	DCIS	10 (1·1%)	13 (1·7%)	0·68 (0·29–1·59)	0·4
	Unknown	0	0	..	..
Contralateral		9 (1·1%)	9 (1·1%)	0·99 (0·39–2·49)	1·0
	Invasive	6 (0·8%)	5 (0·6%)	1·18 (0·36–3·87)	1·0
	DCIS	2 (0·1%)	2 (0·2%)	0·99 (0·14–7·04)	0·8
	Unknown	1	2	..	..
All unknown†		0	2	..	..
All new breast events		29 (4·1%)	33 (5·6%)	0·99 (0·61–1·59)	0·8

Data are number (%). DCIS=ductal carcinoma in situ.

* Laterality or invasiveness unknown.

† Laterality unknown.

**Figure 3:**
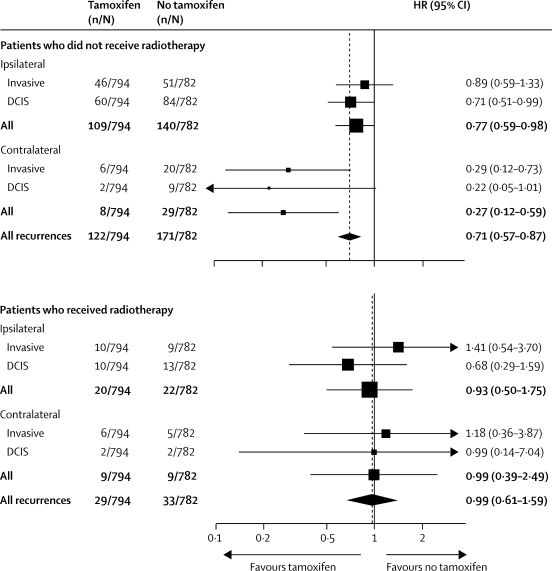
Forest plot for new breast events in the tamoxifen comparison stratified by whether or not patients received radiotherapy HR=hazard ratio. DCIS=ductal carcinoma in situ.

Women who were randomly assigned to tamoxifen but who were not treated with radiotherapy had a significant overall reduction in new breast events (p=0·001), whereas there was no apparent benefit among those who received radiotherapy (p=0·8; table 2; figure 3).

**Figure 4:**
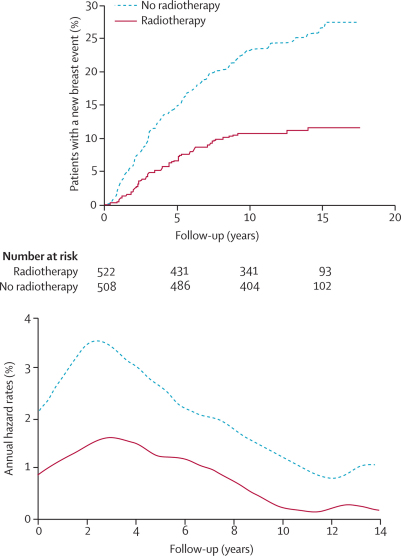
Kaplan-Meier curve for cumulative incidence and annual hazard plot of all breast events in the radiotherapy comparison

**Figure 5:**
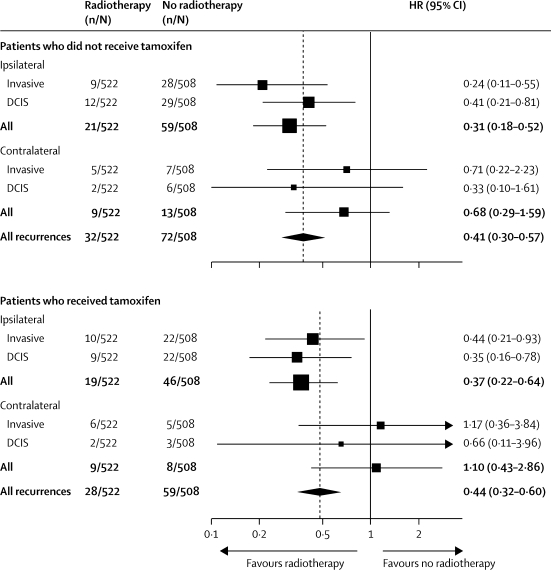
Forest plot for new breast events in the radiotherapy comparison stratified by whether or not patients received tamoxifen HR=hazard ratio. DCIS=ductal carcinoma in situ.

In total, 1030 patients were randomly assigned to receive radiotherapy or not (figure 1). 189 of these patients developed a new breast event: 89 DCIS and 93 an invasive carcinoma (table 3). Overall, those randomised to radiotherapy had fewer new breast events than did those not randomised to radiotherapy (p<0·0001; table 3; figure 4), with an absolute reduction of 12·6%. Radiotherapy significantly reduced all ipsilateral events (p<0·0001) whereas no effect of radiotherapy was reported in relation to contralateral events (p=0·6). In both patients who received tamoxifen and those who did not, a significant reduction in new breast events was reported with radiotherapy (table 3; figure 5).

**Table 3:** New breast events and 10-year estimates of percentages with an event in patients according to radiotherapy randomisation

		**Randomised to radiotherapy (n=522)**	**Randomised to no radiotherapy (n=508)**	**Hazard ratio (95% CI)**	**p value**
**All patients**					
Ipsilateral		40 (7·1%)	105 (19·4%)	0·32 (0·22–0·47)	<0·0001
	Invasive	19 (3·3%)	50 (9·1%)	0·32 (0·19–0·56)	<0·0001
	DCIS	21 (3·8%)	51 (9·7%)	0·38 (0·22–0·63)	<0·0001
	Unknown	0	4	..	..
Contralateral		18 (3·3%)	21 (4·1%)	0·84 (0·45–1·58)	0·6
	Invasive	11 (2·1%)	12 (2·8%)	0·90 (0·40–2·05)	0·8
	DCIS	4 (0·6%)	9 (1·4%)	0·43 (0·13–1·41)	0·2
	Unknown	3	0	..	..
All invasive		31 (5·6%)	62 (10·1%)	0·46 (0·30–0·72)	0·001
All DCIS		26 (4·0%)	63 (11·6%)	0·38 (0·24–0·60)	<0·0001
All unknown*		3	4	..	..
All new breast events		60 (10·6%)	129 (23·2%)	0·41 (0·30–0·56)	<0·0001
**Patients not receiving tamoxifen (n=475)**					
Ipsilateral		21 (4·0%)	59 (11·6%)	0·31 (0·18–0·52)	<0·0001
	Invasive	9 (1·3%)	28 (4·9%)	0·24 (0·11–0·55)	0·001
	DCIS	12 (2·3%)	29 (5·4%)	0·41 (0·21–0·81)	0·01
	Unknown	0	2	..	..
Contralateral		9 (1·7%)	13 (2·8%)	0·68 (0·29–1·59)	0·4
	Invasive	5 (1·0%)	7 (1·0%)	0·71 (0·22–2·23)	0·6
	DCIS	2 (0·4%)	6 (1·0%)	0·33 (0·10–1·61)	0·2
	Unknown	2	0	..	..
All unknown†		2	0	..	..
All new breast events		32 (6·0%)	72 (13·1%)	0·41 (0·30–0·57)	<0·0001
**Patients receiving tamoxifen (n=555)**					
Ipsilateral		19 (3·4%)	46 (8·7%)	0·37 (0·22–0·64)	<0·0001
	Invasive	10 (1·9%)	22 (4·1%)	0·44 (0·21–0·93)	0·03
	DCIS	9 (1·5%)	22 (4·3%)	0·35 (0·16–0·78)	0·01
	Unknown	0	2	..	..
Contralateral		9 (1·5%)	8 (1·4%)	1·10 (0·43–2·86)	0·8
	Invasive	6 (1·1%)	5 (1·0%)	1·17 (0·36–3·84)	0·8
	DCIS	2 (0·2%)	3 (0·4%)	0·66 (0·11–3·96)	0·7
	Unknown	1	0	..	..
All unknown†		1	5	..	..
All new breast events		28 (5·4%)	59 (11·7%)	0·44 (0·32–0·60)	<0·0001

Data are number (%). DCIS=ductal carcinoma in situ.

* Laterality or invasive unknown.

† Laterality unknown.

242 women were randomised to receive tamoxifen and radiotherapy. 25 of these patients developed a new breast event: ten DCIS and 14 an invasive cancer (one unknown). In an analysis restricted to patients in the factorial randomisation, the combination treatment significantly reduced new breast events compared with no adjuvant treatment (p<0·0001). Tamoxifen plus radiotherapy significantly reduced all ipsilateral new breast events (p<0·0001) but had no effect on contralateral new breast events (p=0·2; webappendix p 1). There were no significant differences in new breast events between patients randomly assigned to radiotherapy and tamoxifen and those randomised to radiotherapy alone (webappendix p 2). Patients randomised to radiotherapy and tamoxifen had significantly reduced ipsilateral new breast events compared with those randomised to tamoxifen alone (p<0·0001) but not contralateral new breast events (p=0·5; webappendix p 3).

Tumour blocks were not collected at trial entry, but diagnostic slides have now been collected retrospectively and centrally reviewed for 1224 of the 1694 patients,^16^ which suggested that high grade, large size, and young age were significant predictors of a high recurrence rate.^16^ Table 4 shows details of potential treatment interactions with age and tumour grade. Tamoxifen seemed to be more effective in women with a low-grade or intermediate-grade tumours compared with those with a high-grade tumour. There was a weak effect of radiotherapy in those with intermediate-grade or high-grade tumours. No clear effect of age was seen on tamoxifen efficacy, whereas radiotherapy was more effective in women over 50 years old than in those under 50 years old.

**Table 4:** All new breast events and 10-year estimates of percentages with an event in patients according to grade and age

	**Randomised to tamoxifen**	**Randomised to no tamoxifen**	**Hazard ratio (95% CI)**	**Randomised to radiotherapy**	**Randomised to no radiotherapy**	**Hazard ratio (95% CI)**
**Grade**						
Low (n=105)	2/50 (4%)	11/45 (24%)	0·15 (0·03–0·68)	3/33 (6%)	4/34 (12%)	0·78 (0·18–3·50)
Intermediate (n=267)	12/124 (9%)	24/125 (19%)	0·44 (0·22–0·90)	3/79 (4%)	19/83 (22%)	0·13 (0·04–0·51)
High (n=1014)	112/475 (22%)	138/467 (27%)	0·79 (0·62–1·02)	44/327 (12%)	87/294 (26%)	0·40 (0·27–0·58)
**Age (years)**						
<50 (n=160)	18/77 (22%)	27/69 (35%)	0·58 (0·32–1·07)	13/45 (27%)	16/56 (23%)	0·96 (0·45–2·03)
50–60 (n=919)	87/434 (19%)	102/425 (22%)	0·84 (0·63–1·12)	29/290 (9%)	74/275 (25%)	0·34 (0·22–0·52)
>60 (n=615)	46/283 (16%)	75/288 (25%)	0·59 (0·40–0·85)	18/187 (9%)	39/177 (20%)	0·39 (0·22–0·69)

Data are n/N (%).

179 women had died after a median of 12·7 years of follow-up (table 5). Overall, there was no significant difference in the death rate across treatment groups, but an increase cardiovascular deaths was reported in those randomised to radiotherapy, with or without tamoxifen (p=0·008), although the numbers were small. Deaths from breast cancer seemed to be slightly higher in the tamoxifen group, but this difference was not significant.

**Table 5:** Causes of death

	**No adjuvant treatment (n=544)**	**Tamoxifen (n=567)**	**Radiotherapy (n=267)**	**Radiotherapy and tamoxifen (n=316)**	**Total (n=1694)**
Breast cancer	11 (2%)	19 (3%)	4 (1%)	5 (2%)	39 (2%)
Other cancer	14 (3%)	19 (3%)	10 (4%)	14 (4%)	57 (3%)
Cardiovascular	1 (0%)	3 (1%)	5 (2%)	3 (1%)	12 (1%)
Cerebrovascular	3 (1%)	1 (0%)	0	2 (1%)	6 (0%)
Thromboembolic	3 (1%)	1 (0%)	0	2 (1%)	6 (0%)
Other	20 (4%)	15 (3%)	7 (3%)	17 (5%)	59 (3%)
Total	52 (10%)	58 (10%)	26 (10%)	43 (14%)	179 (11%)

## Discussion

These updated results from the UK/ANZ DCIS trial confirm that radiotherapy significantly reduces the relative risk of ipsilateral new breast events in women with DCIS and suggest that the effect is long lasting (panel). Additionally, these results provide evidence that tamoxifen reduces new breast events in women with locally excised DCIS. The clinically most relevant endpoints are invasive ipsilateral new breast events for radiotherapy because it is a local therapy, and all breast events for tamoxifen because it is a systemic therapy. This is now standard practice, but it was not prespecified in the protocol, which was written in 1989. Designation of these as endpoints of primary interest occurred before the present analyses were undertaken, but this decision was not documented in a formal statistical analysis plan. In our first report,^10^ there was no significant reduction in new breast events with tamoxifen; however, in this long-term follow-up the reduction in new breast events was significant. No effect was identified on ipsilateral invasive new breast events and the largest effect was on contralateral new breast events. The tamoxifen effect seemed to be apparent only in patients who did not receive radiotherapy; however, only 523 patients who received radiotherapy were in the tamoxifen randomisation and a test for interaction between treatments was not significant (data not shown). An effect of tamoxifen was seen in irradiated patients in the only other trial that assessed its use in women with DCIS.^11^

Panel **Research in context**

**Systematic review**

This trial was prompted by the introduction of mammographic screening in the UK, which greatly increased the incidence of ductal carcinoma in situ (DCIS). At that time, three other trials of radiotherapy for DCIS had started (table 6), but no results were available. No trial of tamoxifen for DCIS had started, but there was substantial^19^, ^20^ evidence for its effectiveness in early invasive disease. Two overviews of the trials for DCIS have been published.^13^, ^21^

**Table 6:** Trials of the treatment of ductal carcinoma in situ

	**Entry dates**	**Number randomised**	**Median follow-up (years)**	**HR (95% CI) for ipsilateral new breast events**
**Radiotherapy (50 Gy in 25 fractions recommended)**				
NSABP B-17^7^	1985–90	818	10·7	RR 0·43 (p<0·0001)
EORTC 10853^9^, ^17^	1986–96	1010	10·5	0·53 (0·40–0·70)
UK/ANZ^10^	1990–98	1030	12·7	0·32 (0·22–0·47)
Swedish^18^	1987–99	1067	8·0	0·40 (0·30–0·54)
**Tamoxifen (20 mg for 5 years)**				
NSABP B-24^14^	1991–94	1804	7·0	0·70 (0·50–0·98)
UK/ANZ^10^	1990–98	1576	12·7	0·71 (0·58–0·88)

HR=hazard ratio. RR=risk ratio.

**Interpretation**

This long-term follow-up study confirms the effect of radiotherapy on ipsilateral new breast events and reports an effect of tamoxifen, which was not apparent in the previous analysis. Treatment of DCIS remains challenging because of the low mortality associated with the disease. This trial emphasises the importance of radiotherapy in high-grade DCIS and also suggests a role for tamoxifen primarily for new contralateral disease.

Only 130 (8%) patients were on hormone-replacement therapy at the time of randomisation. Of these, 19 (29·2%) of 65 randomised to tamoxifen developed a new breast event compared with 16 (24·6%) of 65 who were not on tamoxifen. However, we do not have adequate data on the use of hormone-replacement therapy during the trial to be able to reliably draw conclusions from these data.

At present, the NSABP B-24 trial^14^ is the only other study that has assessed the use tamoxifen in DCIS. All women received radiotherapy and tamoxifen significantly reduced recurrences after just over 6 years of follow-up (rate ratio 0·63, 95% CI 0·47–0·83), with weak evidence of a reduction in recurrent DCIS (0·69, 0·46–1·04) and a significant reduction in invasive breast cancer events (0·57, 0·38–0·85). By contrast with our study, a significant reduction in invasive ipsilateral tumours was reported with tamoxifen in these irradiated patients. A possible explanation for the difference is the younger average age of the women in the NSABP study: in our study, over 90% were aged 50 years or more compared with 34% in the NSABP B-17^7^ and B-24^14^ trials.

Our updated findings regarding new breast events from the radiotherapy comparison confirm previous findings.^7^, ^9^, ^10^, ^11^ We noted a slightly larger reduction in ipsilateral new breast events (HR 0·32, 95% CI 0·19–0·56) compared with our previous report (0·38, 0·25–0·59).^10^ The effect of radiotherapy was similar whether or not patients received tamoxifen. The European Organisation for Research and Treatment of Cancer (EORTC) trial^17^ reported updated results on the use of radiotherapy in DCIS in the absence of tamoxifen after a median of 10·5 years of follow-up. A HR for recurrence of 0·53 was reported, which is consistent with the HR of 0·41 we noted in women randomised to radiotherapy who did not receive tamoxifen compared with those who did not have radiotherapy. The NSABP B-17 trial reported the updated results of the effects of radiotherapy after 12 years of follow-up.^11^ The HR for all breast events was 0·59 in women randomised to radiotherapy compared with those who were not. A Swedish study^18^ reported a relative risk of 0·40 for ipsilateral disease in women who received radiotherapy compared with those not irradiated, corresponding to an absolute 10-year reduction of 16%. This reduction is similar to that reported in our study. Furthermore, this study also confirms our finding that older women benefit more from radiotherapy than younger women. The absence of any new breast events in the radiotherapy groups of the UK/ANZ DCIS trial after 9 years (data not shown) differs from the other trials, where the rate appears to be constant, albeit lower than in those not irradiated. This could be a chance finding based on small numbers.

The Early Breast Cancer Trialists' Collaborative Group did a meta-analysis of the effects of radiotherapy on local recurrences and 15-year survival in women with early invasive breast cancer.^22^ Overall, the recurrence rate ratio was about 0·3 in women receiving radiotherapy after breast-conserving surgery compared with those not receiving radiotherapy. This reduction is again similar to that reported in the present analysis.

No significant effects were reported on mortality, either from breast cancer or other causes for either treatment, except for a possible detrimental effect of radiotherapy on cardiovascular deaths, but the numbers were small and further data are needed.

## Web Extra Material

Supplementary webappendix
