# Supplementary material for: Effect of tamoxifen and radiotherapy in women with locally excised ductal carcinoma in situ: long-term results from the UK/ANZ DCIS trial
Source: Lancet Oncol. 2010 Dec 8;12(1):21–9. doi: 10.1016/S1470-2045(10)70266-7 (PMC3018565; doi:10.1016/S1470-2045(10)70266-7)
Supplement: Supplementary webappendix [file mmc1.pdf]

THE LANCET Oncology

Supplementary webappendix

This webappendix formed part of the original submission and has been peer reviewed. We post it as supplied by the authors.

Supplement to: Cuzick J, Sestak I, Pinder SE, et al. Effect of tamoxifen and radiotherapy in women with locally excised ductal carcinoma in situ: long-term results from the UK/ANZ DCIS trial. *Lancet Oncol* 2010; published online Dec 8. DOI:10.1016/S1470-2045(10)70266-7.

	Randomised to radiotherapy and tamoxifen (n=242)	Randomised to no adjuvant therapy (n=226)	Hazard ratio (95% CI)	p value
**Ipsilateral**				
Invasive	9 (3·7%)	27 (10·1%)	0·30 (0·14–0·65)	0·002
DCIS	8 (2·9%)	27 (11·1%)	0·24 (0·10–0·56)	0·001
Unknown	0	2	··	··
**All**	**17 (6·4%)**	**56 (22·7%)**	**0·25 (0·14–0·44)**	**<0·0001**
**Contralateral**				
Invasive	5 (2·1%)	7 (2·8%)	0·69 (0·22–2·17)	0·5
DCIS	2 (0·05%)	6 (2·2%)	0·32 (0·06–1·57)	0·2
Unknown	1	0	··	··
**All**	**8 (2·9%)**	**13 (4·0%)**	**0·59 (0·24–1·41)**	**0·2**
**All invasive**	14 (5·8%)	32 (12·4%)	0·40 (0·21–0·75)	0·004
**All DCIS**	10 (3·3%)	33 (13·4%)	0·25 (0·12–0·52)	<0·0001
Unknown*	1	2	··	··
**All recurrences**	**25 (9·5%)**	**67 (27·0%)**	**0·31 (0·19–0·49)**	**<0·0001**

*Laterality or invasive unknown

Webtable 1: **New breast events and 10-year estimates of percentages with an event in patients randomised to combined treatment or not**

	Randomised to radiotherapy and tamoxifen (n=242)	Randomised to radiotherapy (n=220)	Hazard ratio (95% CI)	p value
**Ipsilateral**				
Invasive	9 (3·7%)	8 (2·7%)	1·37 (0·49–3·84)	0·6
DCIS	8 (2·9%)	11 (5·0%)	0·57 (0·22–1·48)	0·2
Unknown	0	0	··	··
**All**	**17 (6·4%)**	**19 (7·8%)**	**0·85 (0·43–1·68)**	**0·6**
**Contralateral**				
Invasive	5 (2·1%)	5 (2·3%)	0·91 (0·26–3·14)	0·9
DCIS	2 (0·4%)	2 (0·9%)	0·89 (0·13–6·35)	0·9
Unknown	1	2	··	··
**All**	**8 (2·9%)**	**9 (4·1%)**	**0·80 (0·31–2·08)**	**0·7**
**All invasive**	14 (5·8%)	14 (5·5%)	1·06 (0·49–2·28)	0·9
**All DCIS**	10 (3·3%)	14 (5·9%)	0·58 (0·25–1·33)	0·2
Unknown*	1	2	··	··
**All recurrences**	**25 (9·5%)**	**30 (12·4%)**	**0·77 (0·44–1·32)**	**0·3**

*Laterality or invasive unknown

Webtable 2: **New breast events and 10-year estimates of percentages with an event in patients randomised to combined treatment or to radiotherapy alone**

	Randomised to radiotherapy and tamoxifen (n=242)	Randomised to tamoxifen (n=224)	Hazard ratio (95% CI)	p value
**Ipsilateral**				
Invasive	9 (3·7%)	19 (8·5%)	0·43 (0·19–0·95)	0·04
DCIS	8 (2·9%)	19 (8·5%)	0·33 (0·14–0·79)	0·01
Unknown	0	2	··	··
**All**	**17 (6·6%)**	**40 (17·4%)**	**0·35 (0·20–0·62)**	**<0·0001**
**Contralateral**				
Invasive	5 (2·1%)	4 (1·8%)	1·16 (0·31–4·32)	0·8
DCIS	2 [#]	1 [#]	1·85 (0·17–20·46)	0·6
Unknown	1	0	··	··
**All**	**8 (2·9%)**	**5 (1·8%)**	**1·49 (0·49–4·55)**	**0·5**
**All invasive**	14 (5·8%)	23 (10·0%)	0·55 (0·28–1·07)	0·08
**All DCIS**	10 (3·3%)	21 (8·9%)	0·38 (0·18–0·84)	0·02
Unknown*	1	3	··	··
**All recurrences**	**25 (9·5%)**	**47 (19·7%)**	**0·45 (0·28–0·74)**	**0·002**

*Laterality or invasive unknown.

Webtable 3: **New breast events and 10-year estimates of percentages with an event in patients randomised to combined treatment or to tamoxifen alone**
